# Harmonic Generation in Molecular Ag_2_S Plasma

**DOI:** 10.3390/ijms25158106

**Published:** 2024-07-25

**Authors:** Rashid A. Ganeev

**Affiliations:** 1 Institute of Fundamental and Applied Research, TIIAME National Research University, Kori Niyoziy 39, Tashkent 100000, Uzbekistan; rashid.ganeev@lu.lv; 2 Department of Optics and Spectroscopy, Voronezh State University, 394018 Voronezh, Russia

**Keywords:** high-order harmonic generation, laser-induced ablation, Ag_2_S, molecular plasma

## Abstract

The molecular laser-induced plasma (LIP) produced during the ablation of silver sulfide (Ag_2_S) was used as a medium for high-order harmonic generation in the extreme ultraviolet range. The role of LIP formation, the plasma components, and the geometry of plasma in the harmonic conversion efficiency was analyzed. We also analyzed the influence of the driving pulses (chirp, single-color pump, two-color pump, and delay between heating and converting pulses) on the harmonic yield in Ag_2_S LIP. The application of molecular plasma was compared with the application of atomic plasma, which comprised similar metallic elements (Ag) as well as other metal LIPs. The harmonics from the Ag_2_S LIP were 4 to 10 times stronger than those from the Ag LIP. The harmonics up to the 59th order were achieved under the optimal conditions for the molecular plasma.

## 1. Introduction

The applications of lasers interacting with matter for the deposition of thin films [1], the synthesis of nanoparticles [2] and the element analysis of multi-component materials [3] drive expectations that the ablation of materials can also be used for the formation of the sources of coherent short-wavelength radiation through high-order harmonic generation (HHG). HHG in laser-induced plasma (LIP) allows for studying the resonance-induced processes in the extreme ultraviolet (XUV) range, analyzing the role of the morphology of particles (atoms, molecules, clusters, quantum dots, nanoparticles, and microparticles) in the modification of harmonic spectra, demonstrating the quasi-phase-matching of the interacting waves in the periodically modulated LIPs, etc. [4,5,6,7,8,9,10,11,12], which is hard to achieve in the case of other methods of HHG, like harmonic generation in gases [13], from over-dense plasmas (relativistic oscillating mirror process [14] and coherent wake emission [15]), and in solids [16].

HHG in LIP is an effective method for studying the properties of materials [17,18,19,20,21,22,23,24,25,26,27]. The spectral and nonlinear optical properties of ablated materials can be analyzed from the spectra of the generated harmonics while analyzing the relative intensities of some enhanced harmonics along the plateau-like distribution of the generated coherent XUV emission. The first analysis of this peculiarity in the generation of enhanced harmonics in the vicinity of the transition possessing strong oscillator strength was reported in [28]. In this and subsequent studies, the tuning of the harmonic wavelength to an ionic transition possessing strong oscillator strength resulted in a drastic enhancement of the intensity of the single harmonic. Notice that a few of these transitions possessing strong oscillator strength were identified using the NIST tables, while some of the experimental results of the harmonic enhancement pointed out the existence of such transitions, which had been determined earlier.

One such example is the molybdenum LIP. No data were presented in the literature on the strong ionic transitions of Mo in the vicinity of 32.2 nm, which corresponded to the enhanced 25th harmonic of 806 nm radiation [29]. Also, no information is available on the oscillator strengths of the Mo II–Mo IV transitions in this spectral region. Actually, only a few transitions of Mo V are reported in this region. The observation of the influence of the strong transitions in Mo plasma on the harmonic emission demonstrates the advantages of the application of HHG as a tool for the nonlinear optical spectroscopy of molecules, atoms, and ions. Thus, the resonance enhancement of harmonics allows for defining the transitions possessing strong oscillator strengths.

Additionally, the formation of plasma on the surface of the molecular targets greatly depends on the fluence and pulse duration of the heating pulses, as well as on the properties of those targets. Correspondingly, such LIPs can comprise neutral molecules, singly or doubly charged molecules, neutral and charged atomic components of molecules, and free electrons. HHG in LIP allows for determining the presence of these components since their nonlinear optical responses can be distinguished from each other. Moreover, the quasi-phase-matching approach to the enhancement of the group of harmonics in different spectral ranges of XUV allows for determining the concentration of free electrons, which has previously been revealed by laser-induced breakdown spectroscopy.

This method of material science can be further developed by considering the difference in the nonlinear optical response of the molecular plasmas with regard to the atomic one [9,30]. A separate application of the molecules and of the atoms of those molecules being presented in the plasma state can reveal the difference in the generating harmonic spectra (i.e., the harmonic cutoff, the plateau-like shape of the envelope of harmonic distribution, and the harmonic yield), which can be attributed to the specific properties of the atoms and molecules. In this connection, the application of molecular structures like sulfides of metals for HHG and comparison with the harmonic generation in the plasmas containing the metals comprising those molecular structures will provide insight into the usefulness of the molecular plasma for the harmonic generation in the XUV region [31].

In this work, the study of the molecular LIP produced during the ablation of silver sulfide (Ag_2_S) as a medium for HHG is reported. The influence of LIP formation, the role of the plasma components, the geometry of the plasma, and the characteristics of the converting pulses on the harmonic efficiency and harmonic cutoff in Ag_2_S LIP are studied. The application of molecular plasma for HHG is compared with the application of atomic plasma comprising a metallic element (Ag) contained in the silver sulfide.

## 2. Results

### 2.1. Comparison of HHG in Ag_2_S and Ag Plasmas

**Figure 1:**
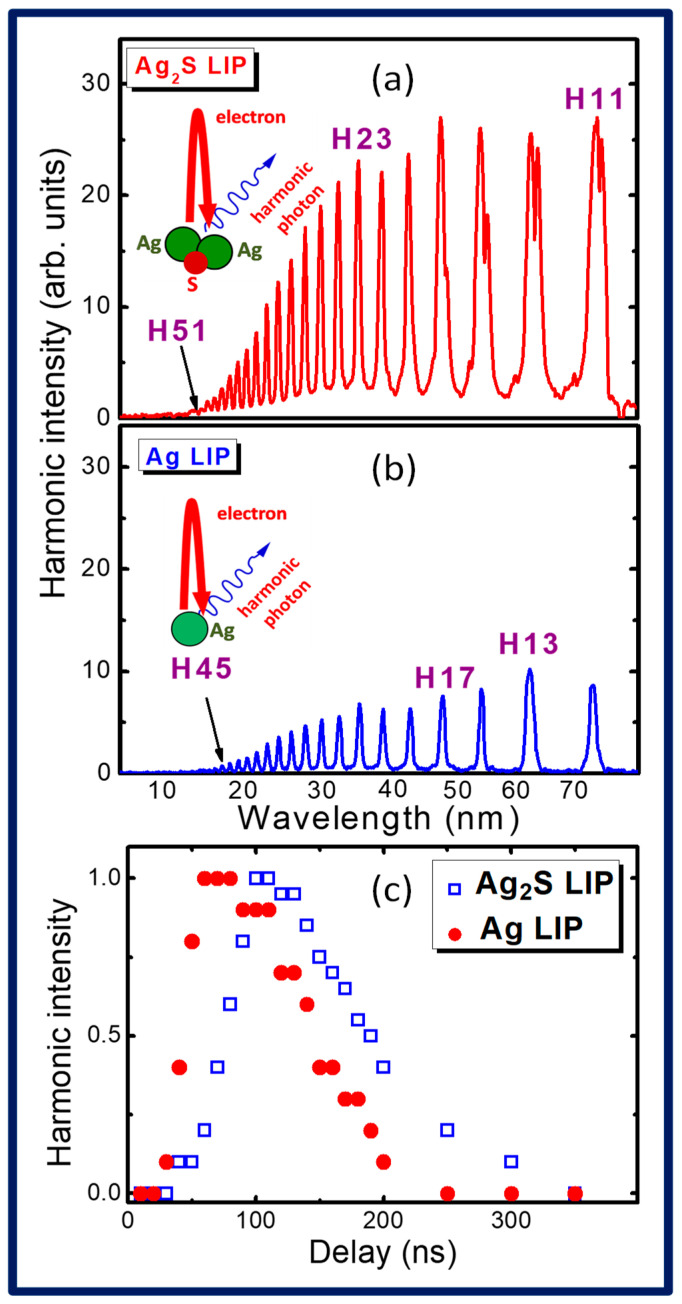
(**a**,**b**) Harmonic spectra generated from the (**a**) silver sulfide and (**b**) silver LIPs. The harmonics up to H51 and H45 were observed. The insets show sketches of the three-step models of HHG from molecules and atoms. (**c**) Dependences of the 19th harmonic yield on the delay between the picosecond and femtosecond pulses in the cases of Ag LIP (filled red circles) and Ag_2_S LIP (empty blue squares).

Figure 1a and Figure 1b presents two harmonic spectra generated in the XUV region (10–80 nm) using silver sulfide plasma and silver plasma, respectively. HHG from silver LIP was chosen for comparison with Ag_2_S LIP due to the similarity of the components and the high conversion efficiency from the former plasma demonstrated in earlier reported studies [32]. The optimization of the plasma formation in the two cases was performed using the variation of the fluence (F) of the heating pulses on the target surfaces and the delay (D) between the heating and driving pulses. In the cases of Ag_2_S and Ag ablations, these parameters were distinguished from each other. Particularly, the optimal F in the cases of silver sulfide and silver plasmas were 0.28 and 0.42 J cm^−2^. The corresponding optimal delays in these two cases were 110 and 70 ns (Figure 1c). In both cases, the choice of F and D was determined at the highest harmonic yield in the plateau-like regions of the harmonic distribution.

The Nth harmonic order is depicted as HN. Particularly, H11 denotes the 11th harmonic order of the 11 × E photon energy, where E = 1.55 eV for the driving pulses at a wavelength of 806 nm. Figure 1a,b show that the harmonics from the Ag_2_S LIP were 4 to 10 times stronger than those from the Ag LIP. The cutoff harmonics from the Ag_2_S and Ag LIPs corresponded to the 51st (H51) and 45th (H45) orders, respectively. 

These spectra were obtained under the conditions of plasma formation (pulse energy of the heating radiation and delay between the heating and driving pulses), which were different for these two plasmas. In the case of the ablation of the bulk Ag_2_S target, the parameters were E_PS_ = 0.2 mJ and D = 110 ns. Similar parameters for the optimally prepared Ag LIP were as follows: E_PS_ = 0.3 mJ, D = 70 ns. The sizes of the ablated areas and the corresponding plasma sizes in both cases were similar to each other (0.3 mm). The collection times of the harmonic spectra by the CCD camera were equal for these two cases. The same holds true for the other experimental parameters, like the distance between the target surface and the driving beam (0.25 mm), the energy of the driving pulses (1 mJ), and the durations of the heating (70 ps) and driving (65 fs) pulses.

The propagation of the plasma cloud out from the target is defined by the kinetic properties of the particles, which can be moved at different velocities depending on the atomic and molecular weight of the ablating material. Different durations from the completion of the target ablation require for the densest part of the plasma to arrive in the area of the driving pulse propagation. Correspondingly, the delay between the heating and driving pulses should be tuned to achieve the maximum yield of harmonics. This delay depends on the elemental composition of the plasma.

One can see that, in the case of the variation of the delays between the heating and driving pulses, the optimal values of this parameter are notably distinguished for these two plasmas (Figure 1c). The maximal yields of harmonics from the Ag atoms and Ag_2_S molecules were observed at 70 ± 10 ns and 110 ± 10 ns delays, respectively. The difference in this parameter was caused by the different velocities of the atoms and molecules spreading out from the target surfaces. The ratio of these delays approximately corresponds to the square root of the ratio of the atomic (108) and molecular (248) weights of the used species. This rule results from the relation between the velocities of particles and their masses in the case of the similar kinetic energies of the species. The highest concentrations of these species arrived at a distance of 0.25 mm from the target surface at different moments from the beginning of the ablation. A gradual decrease in the harmonic yield was observed during the delays exceeding the optimal values. Particularly in the case of H19, the harmonics were observed up to 200 ns and 300 ns delays between the driving and heating pulses in the cases of the Ag atoms and Ag_2_S molecules, respectively.

The ratio of the intensities of the harmonics generated in these two LIPs (I_Ag2S_/I_Ag_) increased with the growth of the harmonic order (Figure 1a,b). This parameter describes the enhancement factor of the harmonics produced from the molecular plasma (Ag_2_S) compared with the harmonics produced from the atomic plasma (Ag). This ratio was equal to 4 in the case of the lower-order harmonics and increased up to 10 close to the cutoff harmonic in the case of Ag plasma (H45). The harmonics up to the 51st order were only generated in the molecular plasma. Thus, the application of the Ag_2_S LIP demonstrated both a stronger yield of harmonics and an extended harmonic cutoff compared with the LIP dominantly comprising the atomic Ag species.

The enhancement of the harmonic yield in the case of molecular LIP plasma can be attributed to the growth in the cross-section of the recombination of the accelerated electron produced during the tunnel ionization of the molecule, which is higher than the cross-section of the recombination of the accelerated electron produced during the tunnel ionization of the atom. The radii of Ag and Ag_2_S particles are ~150 pm and ~350 pm, respectively. Correspondingly, there is a greater probability of an accelerated electron recombining with a larger parent particle (silver sulfide) than with a smaller one (silver). In the former case, the electron can recombine with each atom of the molecule at a larger probability compared with the case of a single atom (see the insets in Figure 1a,b). 

The smaller conversion efficiency in the Ag ablation compared with the Ag_2_S ablation could also be explained by the preferable involvement of ions in the harmonic generation in the former case, while in the case of the ablated molecular target, one can expect the appearance of neutral silver sulfide alongside the molecular ions. The neutral molecules enhance the harmonic emission compared with the ionized species. Thus, the reason for the difference in the harmonic emission observed in the present studies can also be attributed to the difference in the charges of the atoms and molecules in the plasmas under the optimal conditions for laser ablation in the two studied cases.

The fluence of the heating pulses in the case of the Ag_2_S ablation (0.28 J cm^−2^) was optimized from the point of view of the maximal harmonic yield. The effect of the heating pulses on HHG from the ablated Ag bulk target was also observed and optimized using the fluence-dependent yield of harmonics. The harmonic yield and cutoff from the molecular plasma decreased at the heating pulse energies of E_PS_ > 0.25 mJ (F > 0.35 J cm^−2^) due to the growth in the free electron density, leading to the phase mismatch between the interacting waves. The same feature was observed in the case of Ag LIP. These variations in the HHG yield and cutoff confirm that the parameters of the heating picosecond pulses strongly affect this nonlinear optical process in the molecular and atomic medium.

The conditions for optimal plasma formation were determined by the following criteria: Previous estimates of the free electron concentration suggested that this parameter should be in the range of 5–15% with regard to the density of particles in the silver plasma [32]. On the one hand, the strong ablation allows for the formation of denser plasma, resulting in the participation of a larger number of particles in HHG. On the other hand, the increase in the plasma concentration above ~5 × 10^17^ cm^−3^ follows with a drastic increase in the density of the free electrons. These free electrons suppress the harmonic generation in such LIP due to the phase mismatch between the driving and harmonic waves [21].

The optimal conditions for plasma formation for HHG can be determined using laser-induced breakdown spectroscopy, which allows for defining the concentration of free electrons in plasma as well as the density of plasma. The radiative and kinetic properties of LIP, such as the incoherent plasma emission, velocity, and direction of plasma spreading, strongly depend on the plasma and electron parameters (temperature and density), thus influencing the yield of harmonics. The strong plasma emission indicates the conditions for the over-excitation of the ablating target, resulting in the appearance of a large concentration of free electrons in plasma. Additionally, the overlap of this source of radiation emitting within a few nanoseconds with the harmonics produced during a few tens of femtoseconds does not allow separation of the strong incoherent and weak coherent emissions using the time-integrated detectors.

### 2.2. Analysis of Harmonic Modification in Silver Sulfide Plasma Using Variable Driving Pulses and LIP Structure 

The determination of the optimal conditions for HHG requires the analysis of the plasma formation and its characteristics to understand the sequence of mechanisms in this medium. Particularly, the laser-induced atomic plasma formation can be optimized and the harmonic yield can be increased using the estimates of the abovementioned parameters of LIP derived from the hydrodynamic code HYADES. This technique has been demonstrated in the case of the 800 nm driving pulses propagating through the Ag plasma [32]. Meanwhile, further analysis of HHG optimization in molecular plasma requires the application of the variable driving pulses to clarify the relationship between the optimal characteristics of the plasma and the optimal characteristics of the converting radiation. To analyze the latter characteristics, we varied the chirp and duration of the driving pulses. 

**Figure 2:**
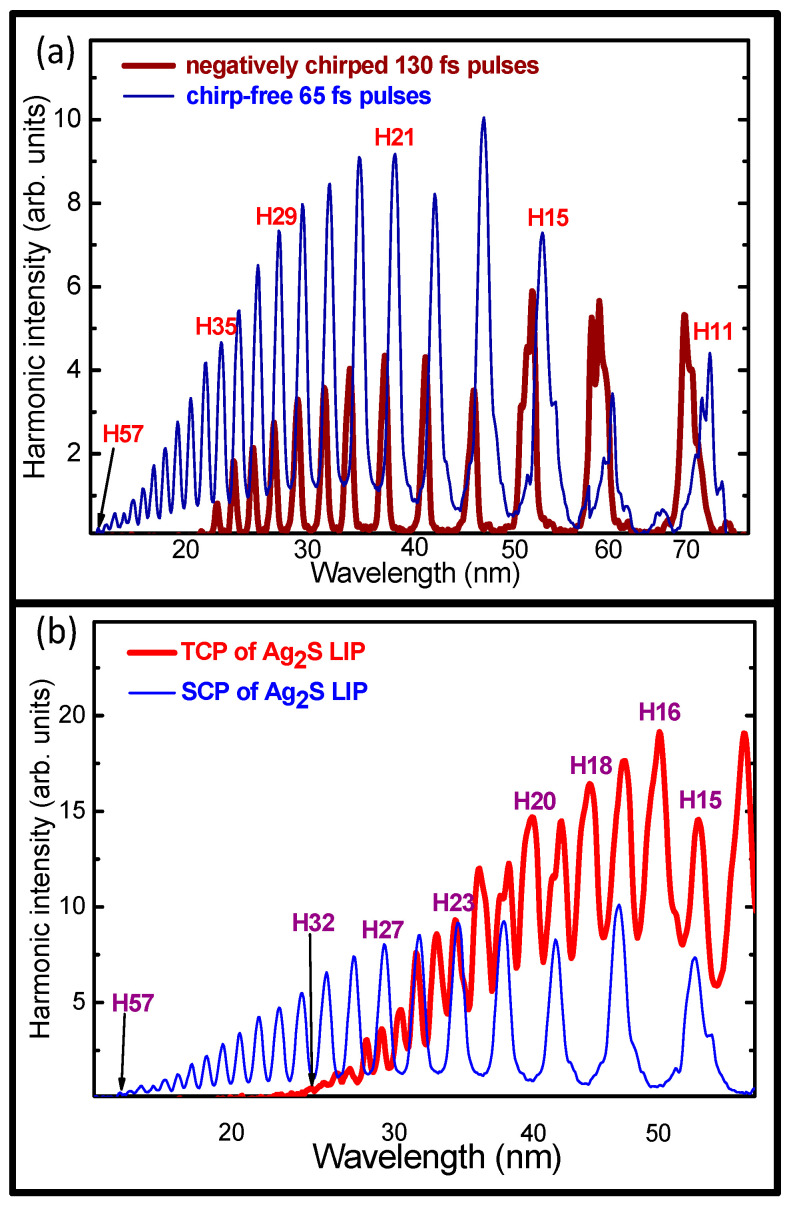
(**a**) Harmonic spectra from Ag_2_S LIP using the chirp-free 65 fs pulses (thin blue curve) and negatively chirped 130 fs pulses (thick brown curve). The following experimental conditions were used: E_FS_ = 1.0 mJ, E_PS_ = 0.2 mJ, and D = 110 ns. (**b**) Harmonic spectra from the Ag_2_S LIP using a single-color pump (SCP, thin blue curve) and a two-color pump (TCP, thick red curve).

Below, we demonstrate the influence of the chirp of the driving pulses on the wavelength of the harmonics produced in the Ag_2_S LIP. The adjustment of the separation between the gratings in the pulse compressor allowed for the chirp variation of the 806 nm radiation. An increase in the grating separation from the chirp-free condition generated the negatively chirped pulses, and a reduction in this distance between the gratings provided the positively chirped pulses. The tuning of the harmonic wavelength during the variation of the laser chirp is shown in Figure 2a. In the cases of the chirp-free and negatively chirped pulses, the featureless plateau-like shapes of the harmonic distributions with a gradual decrease in the harmonic intensity of the higher orders were observed. The intensities of the shorter-wavelength harmonics in the case of the chirped pulses (thick brown curve) were approximately two times smaller compared to the case of the chirp-free pulses (thin blue curve) due to the smaller intensity of the chirped pulses. The duration of these negatively chirped pulses was 130 fs, which was two times longer compared with the chirp-free pulses. Correspondingly, the cutoff in the case of the chirped pulses was expectedly smaller (H35) compared with the case of the chirp-free pulses (H57).

The blue shift of the harmonics produced by the negatively chirped driving pulses depended on the order of the generated emission and varied between 2 nm (H11) and 0.3 nm (H33). The shift in the harmonic wavelength is explained by the wavelength change in the leading edge of the laser pulse during the introduction of the chirp. By varying the chirp of the laser pulse, one varies the spectral components in the leading edge of the pulse. The initial lower-intensity portion of the leading front of the pulse creates harmonics. Correspondingly, only the leading edge of the pulse contributes significantly to the HHG because, with the increase in intensity, the strong field-induced plasma ionization prevents harmonic generation. 

In the case of negatively chirped pulses, the leading part of the driving pulse comprises the shorter-wavelength components of the broadband 806 nm radiation. This facilitates tuning of the harmonic wavelengths toward the shorter wavelength side. Notice that the chirp-induced tuning of the laser spectrum leading to the effective tuning of harmonic wavelength can only be achieved in the case of the broadband pulses. Such a technique has already been explored [33,34,35]. In our case, the pulses used in the present experiments had a bandwidth of ~27 nm, which was sufficient to observe the 2 nm blue-shift of the lowest-order harmonic (H11).

The modification of the interaction of the driving field with matter by adding a weaker, shorter-wavelength source is another method of analyzing HHG in the molecular LIP. We used the single-color pump (SCP, 806 nm) and the two-color pump (TCP, 806 nm + 403 nm) to demonstrate the advantages of the application of the weak second field for the enhancement of high-order odd harmonics and the generation of even harmonics of approximately the same intensity as the odd ones. The insertion of a thin (0.3 mm) barium borate (BBO) crystal in the path of the focused 806 nm beam resulted in a second harmonic generation (403 nm), allowing a sufficient overlap of the two waves in the LIP. The conversion efficiency toward the second harmonic was 6%. The appearance in the present studies of the 4(*n* + 1) even harmonics corresponding to the 16, 20, 24, etc., orders of the 806 nm wave, which cannot be generated by a 400 nm pump, clearly indicates that the two waves properly overlap and interact in the plasma area. 

The relative intensities of the two pumps inside the molecular LIP were determined by the energies of those pulses. Correspondingly, the ratio of the intensities of the 806 and 403 nm pulses in the plasma area was ~16:1. The polarizations of the second and fundamental fields were orthogonal to each other. This difference in the intensities of the orthogonally polarized strong fundamental pulse and the weak second wave did not prevent the generation of almost equal odd and even harmonics in the Ag_2_S LIP (Figure 2b, thick red curve). For comparison, we also show here the harmonic spectrum obtained in the case of SCP (Figure 2b, thin blue curve). The harmonic cutoff in the case of TCP (H32) was predictably smaller than in the case of SCP (H57).

The plots in Figure 2b are presented at similar relative intensities. One can deduce from these plots that the intensities of the low-order odd harmonics in the case of the SCP of Ag_2_S LIP were smaller than the intensities of the odd and even harmonics in the case of the TCP. Thus, the TCP-induced enhancement of the HHG in molecular plasmas showed that the addition of a weak second wave allows for the generation of odd harmonics, which are stronger than those obtained using the fundamental field alone. Moreover, the TCP allowed for simultaneously generating even harmonics as strong as the odd ones, which led to the increase of the HHG conversion efficiency. This strong harmonic generation in the case of a two-color field inside the molecular LIP can be explained by the selection of a short quantum path component, which has a denser electron wave packet and a higher ionization rate compared with the SCP. 

The geometry of plasma formation also plays an important role in the modification of the harmonic distribution along the plateau-like region. Most of the present studies were performed using the narrow molecular plasma (~0.3 mm) produced during the focusing of the heating picosecond pulses by the spherical lens. Meanwhile, we also analyzed HHG in the molecular plasma produced during the ablation of silver sulfide using a cylindrical lens. In that case, the 5 mm long plasma was formed. The growth of the harmonic yield in the extended plasma competes with the reabsorption of the generated harmonics in the elongated plasma and the enhanced phase mismatch accumulated during the propagation of the driving pulses through the extended medium. The role of the free electrons in the case of the 0.3 mm thick plasma was insignificant for both the low- and high-order harmonics. Meanwhile, in the case of extended plasma, the accumulative effect of the free electrons led to the suppression of the highest orders of harmonics. 

**Figure 3:**
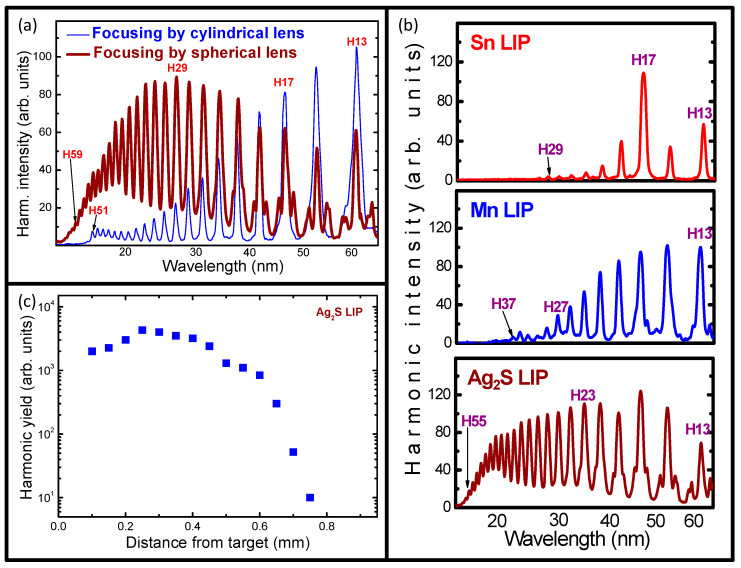
(**a**) Harmonic spectra from the Ag_2_S LIPs in the cases of the 0.3 mm long (thick brown curve) and 5 mm long (thin blue curve) plasmas. (**b**) Harmonic spectra generated from the Sn (**upper** panel), Mn (**middle** panel), and Ag_2_S (**bottom** panel) plasmas. (**c**) Dependence of the 13th harmonic yield on the distance between the Ag_2_S target and the femtosecond beam.

The heating picosecond pulses were focused using the 200 mm focal length cylindrical lens inside the vacuum chamber containing an ablating target to create the extended plasma plume. The intensity of the heating 70 ps pulses on the target surface at these conditions of focusing was 4 × 10^9^ W cm^−2^ (E_PS_ = 4 mJ) to maintain the same fluence as in the above-described studies (0.28 J cm^−2^). The plasma sizes were 5 × 0.3 mm^2^. The focused driving femtosecond beam (confocal parameter 8 mm) propagated through this extended plasma. Figure 3a shows a gradual decrease in the higher orders of harmonics in the case of the extended Ag_2_S LIP (thin blue curve), which was contrary to the harmonic distribution in the case of the narrow plasma (thick brown curve). Under the conditions of weak absorption and the insignificant influence of the mismatch effect, the unsaturated harmonic yield should follow the quadratic dependence on the length of the nonlinear medium *I*_H_ ∞ (*l*_plasma_)^2^. However, exceeding the length of the plasma over the coherence length of some harmonics should lead to saturation and a decrease in the yield of the higher-order harmonics. Notice that even a small mismatch between the interacting waves introduced by free electrons, which do not play an important role in the case of the small sizes of LIP, becomes accumulated along the 16-times longer plasma. This accumulative effect causes a preferential decrease in the harmonic yield for the highest orders.

Earlier, the application of extended Ag_2_S plasma allowed for the demonstration of the quasi-phase-matching effect when the modulation of the extended plasma resulted in the enhancement of the higher-order harmonics while suppressing the lower-order ones. The installation of the multi-slit mask between the cylindrical lens and the Ag_2_S target allowed for the formation of the multi-jet plasma instead of the extended imperforated LIP [36]. The application of this molecular plasma was also reported in [30]. It was shown that the quasi-phase-matching conditions in the case of the multi-jet Ag_2_S LIP were less efficient compared with the quasi-phase-matching conditions created using the perforated silver plasma.

The HHG in Ag_2_S LIP was compared with similar processes in two metal plasmas (Sn and Mn). The corresponding harmonic distributions are shown in Figure 3b. In these two cases, similar to the case of Ag LIP (Figure 1b), the HHG conversion efficiencies in the Sn and Mn LIPs were lower compared with the Ag_2_S plasma.

Finally, we analyzed the harmonic yield at different distances from the Ag_2_S surface. Apart from the density and the degree of excitation of the neutral molecules and singly charged ions, the location of the focusing area of the driving beam at the appropriate distance from the target allows for efficiently matching the spreading of the plasma and the propagation of the converting radiation. Figure 3c shows a sharp decrease in the 13th harmonic yield once the distance between the target and the focal spot becomes larger. The largest yields of harmonics were obtained in the 0.25–0.3 mm range of this parameter at the used delay between the heating and driving pulses (110 ns). A decrease in the plasma density at longer distances from the target causes a significant drop in the HHG conversion efficiency.

## 3. Discussion

The benefits of generating high-order harmonics in LIP include the formation of coherent XUV sources, which can be used for various applications. Though HHG is established as a high-output coherent light source in the XUV region, it is also the sole source of attosecond pulses. This radiation can also be used in traditional applications in atomic, molecular, and solid-state physics, such as lifetime measurements. Coherent light sources based on HHG in gases and plasmas can be employed in a broad range of subjects, including basic research, material science, biology, and lithography [37]. Furthermore, the HHG process in molecules encodes electronic orbital structure information and presents, as a consequence, a reliable method to retrieve molecular intrinsic parameters. 

The novelties of the present research with regard to previous reports related to the molecular and atomic LIPs [30,31,32,36] are as follows. In [30], the HHG in LIP was analyzed from the point of view of the presence of different molecular and atomic components in these plasmas. Particularly, the harmonic yields from the carbon and boron plasmas were stronger than those from the boron carbide plasma. Additionally, the harmonic cutoffs from the atomic and molecular plasmas were significantly distinguished from each other (H57 and H23 in the case of B and B_4_C LIPs, respectively, while using the 800 nm driving pulses). Contrary to that, in the present study, the harmonics from the Ag_2_S LIP were 4 to 10 times stronger than those from the Ag LIP, and the corresponding cutoff harmonics were of the 51st order (H51) and the 45th order (H45), respectively, i.e., the reverse case was observed with regard to the above-mentioned study. This result points out the importance of considering different components of molecules with regard to the molecule as the sources of harmonics to determine the best conditions for HHG in each specific case. The next reference [31] analyzed the quasi-phase-matching in the molecular plasmas, which was not a topic of the present research. Additionally, the referenced study demonstrated the conditions for molecular target ablation when the resonance-induced processes started to play a decisive role in the enhancement of a single harmonic. In the present study, the resonance processes were not considered due to the absence of the strong ionic transitions and resonance enhancement in the silver-contained plasmas. The goal of the studies reported in [32] was the analysis of the influence of the heating and driving pulses on the harmonic spectrum obtained from silver ablation, which was out of the main scope of the present study of the molecular (Ag_2_S) LIP. Finally, the research reported in [36] concentrated on the HHG using the SCP and TCP of extended (5-mm-long) plasmas produced on the surfaces of various atomic materials using different sources of second harmonics. In the present study, the two-color pump is used to demonstrate the advantages and disadvantages of this method of HHG in the case of the molecular plasma (Ag_2_S).

Summing up, previous studies demonstrated the worsened characteristics of the generated coherent XUV radiation in the molecular LIP (weaker conversion efficiency, lower orders of generating harmonics, insufficient stability, weakened quasi-phase-matching effect, and absence of the resonance-induced enhancement of single harmonic). Meanwhile, our studies allow the conclusion that application of simple species like neutral atoms or singly charged atoms as the media for HHG in some cases, like silver-contained targets, demonstrates lesser conversion efficiency and harmonic cutoff over molecular species. To prove this conclusion, various processes influencing the efficiency of HHG in silver sulfide plasma (characteristics of LIP formation, role of plasma components, geometry of plasma, delay between heating and converting pulses, chirp variation, single- and two-color pump) were analyzed, resulting in the optimization of this process and generation of the harmonics up to the 59th order. It was shown that the harmonics from the Ag_2_S LIP were 4 to 10 times stronger than those from the Ag LIP. Thus, our study demonstrates that this molecular plasma is an effective medium for harmonic generation in the XUV region. The additional differences between the present study and previous reports are related to (a) the inclusion of the analysis of the harmonic yield at different positions of the plasma cloud with regard to the target; (b) comparison of Ag_2_S LIP and atomic plasmas (Sn, Mn), demonstrating the non-resonance and resonance enhancement of the harmonics; (c) analysis of narrow and extended molecular plasmas as the media for the generation of variable distributions of harmonics; (d) joint demonstration of the advantages and disadvantages of the TCP of molecular plasma; and (e) tuning of the harmonics produced in the molecular LIP using the chirped pulses.

Below, Table 1, which summarizes the studies of HHG in Ag_2_S under optimal conditions for the experiments, is presented. 

## 4. Methods and Materials

The plasma on the surface of the Ag_2_S plate (98%, Sigma-Aldrich, Burlington, MA, USA) placed in a vacuum chamber was formed by a 70 ps, 1064 nm, 10 Hz emission using a picosecond Nd:YAG laser (PL2250, EKSPLA, Vilnius, Lithuania). The bulk metals (Ag, Mn, and Sn) were also applied for plasma formation to carry out a comparative analysis of HHG from the molecular (Ag_2_S) plasma and atomic plasmas comprising the metal species.

**Figure 4:**
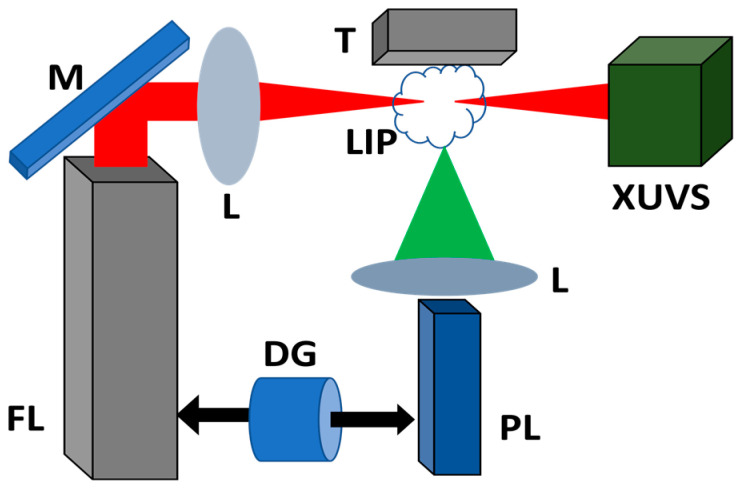
Experimental scheme for harmonic generation in laser-induced plasmas. PL: picosecond laser (70 ps, 1064 nm) for plasma ablation; FL: femtosecond laser (65 fs, 806 nm) for harmonic generation; DG: digital delay generator; M: mirror; L: lens; T: target; LIP: laser-induced plasma; XUVS: XUV spectrometer.

After some delay from the beginning of plasma formation, the driving pulses (65 fs, 806 nm, 10 Hz, 1 mJ) from the femtosecond laser (TSA-10, Spectra-Physics Lasers, Tokyo, Japan) were focused inside the laser-induced molecular or atomic plasmas (Figure 4). The delay between the heating and driving pulses was accomplished using a digital delay generator, allowing for the variation of this parameter along a broad range (0–10,000 ns). For each case of plasma formation, the optimal delay was determined, which allowed for the generation of the maximal harmonic yield. The term “optimal delay” refers to the conditions of HHG experiments in LIP when the maximal yield of harmonics generates at a specific time difference between the ablation by the heating pulses and the propagation of the driving pulses above the target surface. This specific time difference determines the arrival of the maximal density of ablated particles in the area of propagation of the driving pulses. Correspondingly, the most efficient conditions for harmonic generation are established at this delay of driving femtosecond pulses with regard to the heating picosecond pulses.

The intensity of the driving femtosecond pulses focused inside the LIP was maintained at *I* = 3 × 10^14^ W cm^−2^ during the whole set of experiments. The distance between the targets and the optical axis of propagation of the driving femtosecond beam (d) was adjusted using the translating stage. Most of the experiments were carried out at d = 0.25 mm. 

The plasma and harmonic emissions in the XUV range were analyzed by an extreme ultraviolet spectrometer (XUVS, Figure 4), which contained a gold-coated cylindrical mirror and a 1200 groove/mm flat field grating (FFG, 124, Hitachi Photonics, Ibaraki, Japan) with variable line spacing. The harmonic emission was recorded by a micro-channel plate (MCP, F2813-22P, Hamamatsu, Iwata City, Japan) with a phosphor screen, and the XUV spectra were collected using a CCD camera (C4880, Hamamatsu, Iwata City, Japan). The measurements of the harmonic spectra were carried out in a time-integrated mode.

## 5. Conclusions

Laser-induced molecular (silver sulfide) plasma was used as a medium for HHG of the 806 nm, 65 fs pulses. Various processes influencing the efficiency of HHG (characteristics of LIP formation, role of plasma components, geometry of plasma, delay between heating and converting pulses, chirp variation, single-color pump, two-color pump) were analyzed, resulting in the optimization of this process and generation of the harmonics up to the 59th order. The application of this molecular plasma for HHG was compared with the application of the atomic plasma comprising similar metallic elements (Ag) as well as other metal LIPs. It was shown that the shorter-wavelength harmonics from the Ag_2_S LIP were 4 to 10 times stronger than those from the Ag LIP. These studies demonstrated that this molecular plasma is an effective medium for harmonic generation in the XUV region. The perspectives of the present studies include the application of the approaches used for the enhancement of the harmonic yield from the molecular plasmas, especially those that allow generation of the resonance-enhanced harmonics (i.e., molecules comprising Mn, In, Cr, Te, Sn, and Zn atoms). 

## Figures and Tables

**Table 1:** Optimal parameters of HHG experiments using silver sulfide plasma.

Optimal Delay	Ratio of Intensities (I_Ag2S_/I_Ag_) from Ag_2_S and Ag LIP	Optimal Fluence of 70 ps Heating Pulses	Intensity of 806 nm Driving Pulses	Harmonic Cutoff	Harmonic Shift for Chirped Driving Pulses	TCP-Induced Enhancement of Low-Order Harmonics	Optimal Distance from Target	Ratio of Harmonic Intensities (I_5mm Ag2S_/I_0.3mm Ag2S_) Using LIPs of Different Length
110 ns	4 (H11) 10 (H49)	0.28 J cm^−2^	3 × 10^14^ W cm^−2^	H59	2 nm (H11) 0.3 nm (H33)	2×	0.25 mm	2 (H13) 0.15 (H51)

## Data Availability

The data that support the findings of this study are available on request from the corresponding author. The data are not publicly available due to collecting in the computer.
